# Cell consciousness: a dissenting opinion: The cellular basis of consciousness theory lacks empirical evidence for its claims that all cells have consciousness

**DOI:** 10.1038/s44319-024-00127-4

**Published:** 2024-03-28

**Authors:** David G Robinson, Jon Mallatt, Wendy Ann Peer, Victor Sourjik, Lincoln Taiz

**Affiliations:** 1 Centre for Organismal Studies, University of Heidelberg, Heidelberg, Germany; 2 WWAMI Medical Education Program, University of Idaho, Moscow, ID USA; 3 Department of Environmental Science and Technology, University of Maryland, College Park, MD USA; 4 Department of Systems and Synthetic Microbiology, Max Planck Institute for Terrestrial Microbiology, Marburg, Germany; 5 Department of Molecular, Cell, & Developmental Biology, University of California, Santa Cruz, CA USA

**Keywords:** History & Philosophy of Science, Neuroscience, Plant Biology

## Abstract

The proponents of CBC claim that all living organisms down to prokaryotes have consciousness. However, their arguments lack empirical evidence or are refuted by established facts.

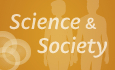

Consciousness is a mental property, specifically the ability to experience feelings that yield a subjective point of view (see “Definitions” in Box 1). The mechanisms of consciousness remain elusive, but converging evidence from studies in humans and animals shows crucial contributions of several brain regions and functional states (Koch et al, 2016). Injuries that affect critical brain regions can induce profound alterations in consciousness, or, in the case of being in a coma, extended periods or permanent loss of consciousness (Alnagger et al, 2023). Conversely, direct electrical stimulation of these brain regions can elicit various sensations, perceptions and mental experiences (Penfield, 1958). In a recent groundbreaking experiment, a song by Pink Floyd was successfully reconstructed from the recordings of brain activity of the individuals listening to it (Bellier et al, 2023). This established the specific site of music perception in the brain.

Together, these studies would seem to make the case that the brain is the seat of consciousness in animals a, well, no-brainer. Accordingly, most neuroscientists and experimental psychologists accept the paradigm that the evolution of consciousness required the development of a nervous system and a brain of threshold complexity, perhaps sometime during the Cambrian explosion about 500 million years ago in association with the onset of predator-prey interactions (Taiz et al, 2019). Consequently, consciousness exists in all vertebrates, arthropods and cephalopod mollusks such as octopuses and squids.

… most neuroscientists and experimental psychologists accept the paradigm that the evolution of consciousness required the development of a nervous system and a brain of threshold complexity …

Nevertheless, some investigators remain unconvinced. An alternative view, termed the Cellular Basis of Consciousness (CBC), was proposed by the psychologist Arthur Reber in 2016, and subsequently elaborated upon by Reber and his colleagues (Baluška and Reber 2019). The most recent version (Reber et al, 2023) was derived from a parent book by these authors called *The Sentient Cell*; 2023, Oxford University Press. According to the CBC model, consciousness and cognition are fundamental properties that evolved with the first prokaryotic cells around 3.8 billion years ago. This controversial theory, for which no plausible mechanism has been provided, raises many unaddressed issues. Indeed, the central criticism of CBC has been that it is based on an elaborate series of speculations for which empirical evidence is lacking (Mallatt et al, 2021). CBC’s proponents admit to this approach by calling it “educated speculation” (Baluška and Reber 2019).

… the central criticism of CBC has been that it is based on an elaborate series of speculations for which empirical evidence is lacking.

## CBC’s terminology and the types of consciousness

While remaining speculative, the CBC theory recently took on two new aspects that raise further questions. First, Reber et al now feel there is too much disagreement about the definition of consciousness for it ever to be defined, so they instead characterize consciousness with a variety of interchangeable “synonyms and near-synonyms.” Thus, their characterization synonymizes “sentience” with “consciousness” with “cognition” and with “intelligence.” Although these terms are related, the key distinctions among them (see “Definitions” in Box 1) are lost by conflating them. For example, an entity can carry out the information processing of cognition but lack the experienced feelings of consciousness. Their synonymizing and interchanging of different terms was purportedly done to “prevent misunderstanding,” but it does the opposite by making the terminology imprecise.

Second, Reber et al have expanded their characterization of cell consciousness. The CBC theory originally said that cells have only the most primitive or basal form of consciousness, but now it assigns more abilities to cells, including types of *higher* consciousness (see “Definitions” in Box 1). These higher types are underlined in the following quotation: “We regard consciousness as a cohesive suite of cognitive and affective faculties accompanying valenced experiences, self-referencing, self-awareness, organized information assessment, [conscious] communication, learning, memory, decision-making …” (p. xii in the parent book). The claim that all cells have higher consciousness made the CBC increasingly radical and unorthodox and raises a host of questions that are even more difficult to test empirically.

Box 1 Definitions **A. Cognition and consciousness**. These are difficult terms with various definitions, none of which are universally accepted. However, there is enough agreement that both terms can be generally understood and intelligently discussed.

**Cognition**: Though this term is linked to thinking and knowing in humans, both we and the proponents of CBC can accept a broader definition from the comparative psychologist Sara Shettleworth: the ability to receive, process, store and act on information from the environment, including memory, learning and making decisions (Taiz et al, 2019; Reber et al, 2022).

**Consciousness:** In the field of consciousness studies, definitions converge on consciousness being the capacity to have feelings or experiences, yielding a subjective or first-person point of view (Mallatt et al, 2021).

**Basic type**: The most basic form is **phenomenal (primary) consciousness**, which is raw experiences, in-the-moment, and not reflected upon. This has several aspects: experiencing what one senses (“I see a colorful sunset”) and experiencing affects (feeling emotions and moods). These two aspects comprise **sentience** (Mallatt et al, 2021). Another aspect of phenomenal consciousness is feeling the desire to act, so it signals voluntary actions.

**Higher types** can exist in addition to phenomenal consciousness. They include the self-awareness that reveals one is conscious, recognizing oneself in a mirror, realizing that others have consciousness (theory of mind), episodic memories of one’s past personal experiences, mental time-travel, and abstract thinking. Humans have these types of consciousness, and an important question is whether other organisms do.

**B. Other major theories that focus on human consciousness**:

**Global neuronal workspace** (of Stanislaus Dehaene, Bernard Baars, etc.): Our sensory perceptions, emotions and memories are processed locally in various regions of our brain’s cerebral cortex, but they only become conscious when strong or salient enough to enter a broad, central neural network called the global workspace. The salient item “ignites” this network and reverberates widely over the frontal and parietal cortex.

**Recurrent processing** (of Victor Lamme and others): Sensory information such as visual input from the retina feeds forward in our cerebral cortex through successively higher levels of processing. For this input to become conscious, however, requires extensive feedback from the higher to lower regions of the hierarchy, resulting in ongoing feedback loops.

**Integrated information theory** (of Giulio Tononi and others): Every system whose parts interact has integrative information and therefore consciousness. Even simple, nonliving systems have some consciousness. However, the theory deals heavily with the quantity and quality of information integration in the human cerebral cortex.

**Higher-order theories:** This diverse set of theories says that basic perceptions of what we sense only become conscious when these “lower-order representations” are re-represented at higher levels in the brain; that is, when the percepts become available to higher-order thought.

**Predictive coding theories** (of Karl Friston and others): The brain is constantly forming predictions about the incoming sensory signals and updating these predictions based on the actual sensory input, continually lessoning the errors in the predictions. Consciousness should be understood in terms of these fine-tuned predictions.

## Can CBC theory validly dismiss brains and humans?

All the standard theories of consciousness stipulate that it is limited to multicellular organisms possessing neurons and a brain of threshold complexity (Francken et al, 2022). Reber et al (2023), on the other hand, assert that all the requirements for consciousness, including the capacity to have experiences, to feel pain and to act intentionally, reside in individual cells. The CBC theory has been criticized on the grounds that it has not explained the many *unconscious* processes the nervous system performs—motor programing, reflexes, subliminal perception and so on—which would be impossible if each individual nerve cell were conscious (Morsella and Reyes, 2016).

Reber et al (2023) criticize the standard theories of consciousness for being too human-oriented. They argue that because humans have the most sophisticated and highest types of consciousness, humans are not a reliable guide to the phylogenetic origin of consciousness in its simplest form. This argument has some validity because most standard theories of consciousness are strongly human-oriented. These theories are listed and briefly explained in Part B of Box 1 as the global neuronal workspace, recurrent processing, integrated information, higher order, and predictive coding theories. Yes, their human-centered perspective could conceivably bias against recognition of conscious species that are unlike us.

However, Reber et al should not have dismissed human consciousness so fully. Humans undoubtedly have consciousness, so they must be included as a reference point. In fact, without referring to human experience, there can be no direct knowledge for defining sentience. Human-derived information on consciousness has informed tests that credibly demonstrate consciousness in other primates such as rhesus monkeys (*Macaqua mulatta)*, in dolphins like *Tursiops truncatus*, and in crows like *Corvus corone*. The best way to reconcile the human-bias problem is to recognize that besides the higher types of consciousness, humans also have basic phenomenal consciousness (see “Definitions” in Box 1). To minimize bias, one can employ functional and anatomical markers of this most basic form of consciousness in humans to investigate whether they also occur in other organisms (Ehret and Romand, 2022).

To minimize bias, one can employ functional and anatomical markers of this most basic form of consciousness in humans to investigate whether they also occur in other organisms.

## Consciousness and the single cell

Reber et al (2023) begin their argument that all living organisms are conscious and self-aware by referencing unicellular prokaryotic organisms, which “display behaviors that are clearly cognitive in nature.” There are several major leaps in this argument, and much of it is based on the misinterpretation of research on bacterial sensory responses and behavior. First, as mentioned above, they equate cognitive with conscious and these two may not be the same. Even if some prokaryotic behaviors might be cognitive, this cannot be taken as evidence for their consciousness. Second, not every response to environmental stimulation needs to be classified as cognitive, even if we accept a very broad definition of it. This is particularly true for prokaryotic organisms, which in many cases rely on simple two-step phosphorylation pathways to convert environmental stimuli into changes in gene expression. Although the idea that bacterial sensory systems might be integrated into an information processing network had been discussed in the early days of bacterial systems biology (Hellingwerf et al, 1995), subsequent studies have shown that most individual sensory pathways operate independently, without a common decision-making process (Laub and Goulian, 2007).

Yet, in some instances, bacteria do appear to exhibit behaviors that resemble those we normally associate with cognitive behaviors in animals. The most prominent example, which Reber et al (2023) cite as “…memory formation, route navigation and decision-making,” is bacterial chemotaxis. Indeed, chemotaxis is a remarkable system that enables motile bacteria to navigate in the environmental gradients of chemical and other stimuli, including making “decisions” where to go in conflicting gradients of stimuli (Colin et al, 2021). This behavior relies on a short-term “memory” about the past to enable it to perform temporal comparisons of changes in the environment. Bacteria can even make “anticipatory” adjustments in the allocation of cellular resources for motility and chemotaxis that depend on the potential benefit they may receive from chemotaxis in a particular environment (Colin et al, 2021).

All of this could give the impression of a “conscious” behavior. However, to call it conscious ignores fundamental differences between decision-making in prokaryotes and animals. In prokaryotes, all of these “decision-making” behaviors are hardwired in their genome as rates of biochemical reactions and levels of corresponding enzymes. The entire decision-making process that underlies bacterial route navigation, including choices of direction, can be broken down to a few individual molecular reactions described by a relatively simple system of differential equations (Colin et al, 2021) leaving no place for consciousness or volitional decisions. An individual bacterial cell does not make a choice—the decisions are determined by its current state; and even when individual cells behave differently, it can be traced to stochastic differences in protein levels between cells.

An individual bacterial cell does not make a choice—the decisions are determined by its current state…

The same is true for the reported anticipatory responses displayed by prokaryotes, which presumably led Reber et al (2023) to conclude that prokaryotes “display … associative learning.” There is no convincing evidence for associative learning by prokaryotic cells (Perkins and Swain, 2009). Instead, the known anticipatory behaviors displayed by bacteria are products of Darwinian natural selection, where environments with frequently co-occurring parameters—such as oxygen and temperature—select for random mutations that happen to couple particular responses (Tagkopoulos et al, 2008). Hence, the association in this case is evolutionarily genetically hardwired and has nothing to do with learning by individual organisms and cannot be taken as evidence for conscious or even cognitive behavior.

Other behaviors seemingly analogous to those that we normally associate with cognition and/or consciousness in humans can be readily identified in prokaryotes. But a closer inspection demonstrates that these commonalities have different causes. For example, *Pseudomonas aeruginosa* are known to respond chemotactically to GABA (Reyes-Darias et al, 2015). However, their chemotaxis towards GABA has nothing to do with its neurotransmitter activity or cognition. Instead, GABA is recognized by a specific chemoreceptor of these soil-dwelling bacteria as one of the multiple signals they use to colonize and infect the plant roots that release it.

## Do plants anesthetize themselves to avoid pain?

Reber et al (2023) state unequivocally that “plants …. are conscious, sentient beings.” This is a bold claim, considering that the opposite has been concluded in several publications that they fail to cite (Taiz et al, 2019; Mallatt et al, 2021). In support of their claim, Reber et al (2023) simply say that “there is considerable evidence for valenced [that is, emotionally felt, such as “appealing” or “repulsive”] sensation, decision-making, learning, and communication in flora.” Of course, plants can detect and react to environmental cues, but these are genetically programmed responses mediated by physiological adaptations that do not require higher-order psychological concepts to explain. During herbivory, plants can also emit volatile defense compounds that can evoke defense responses in neighboring plants, a limited form of “chemical communication” that is strictly unidirectional. However, plants are not capable of making true anticipatory decisions, nor do they possess the facility for memory-based learning of the operant type that could indicate consciousness. In fact, it is even debated whether they can learn by simple, unconscious, Pavlovian conditioning (Ponkshe et al, 2023).

Plant biologists have often pointed to the existence of neurotransmitters like glutamate and GABA as indicators of cognition/consciousness in plants. However, in evolutionary terms, both substances evolved prior to nervous systems, and their receptors appear to be localized on the endomembranes rather than plasma membranes of plants. Instead of functioning in cognitive processes, their role appears to be in hormone signaling (Robinson et al, 2023; and above).

It is well known that plants are sensitive to anesthetics, and that the effects are reversible (Robinson et al, 2023). This observation has been repeatedly interpreted by plant neurobiologists as indicating that plants feel pain and thus are sentient. Reber et al (2023) ask rhetorically (p. 3), “If a species doesn’t feel pain why be sensitive to compounds that block the experience of pain?” Citing the plant origin of many anesthetics, they suggest that the anesthetics released from plants upon wounding must have a “stress-relieving” effect on the plant itself. This conclusion is demonstrably false. Since the principal targets for anesthetics in humans are ion channels—ligand-gated receptors for glutamate and GABA as well as voltage-gated K + -channels—and acetylcholine receptors, but also microtubules (Kelz and Mashour, 2019), it is not surprising that plant membranes (and cells), which have these same elements, will be affected by anesthetics as animal cells are. That plants also synthesize the amino acid glutamate and GABA-like proteins is irrelevant to an understanding of anesthetic action on plants.

While many anesthetics are of plant origin, the plants synthesize them for many functions but lack the animal anesthesia targets that would provide relief from pain or stress. These anesthetics are the products of a “secondary” metabolism that is unique to plants and serves to produce defense compounds that deter and inhibit predation and prevent infection. These defense compounds are also allelopathic and inhibit metabolism, acquisition of nutrients, and cell division to reduce competition from other plants. Some defense compounds are constitutively present while the synthesis of others can be induced or activated by herbivory, infection and abiotic stress.

While many anesthetics are of plant origin, the plants synthesize them for many functions but lack the animal anesthesia targets that would provide relief from pain or stress.

The majority of these toxic chemicals, such as the alkaloids caffeine, nicotine, cocaine and atropine are stored in the vacuole, and the remainder are stored in specialized cell types that are “fire-walled” from living plant cells. Other alkaloids such as codeine and morphine are synthesized and stored in phloem laticifers, and when a herbivore breaks the laticifers, these analgesic compounds are released and act as a sedative to animals/insects to prevent further predation. Caffeine, nicotine, cocaine and atropine that function as anticholinergics block acetylcholine binding in animal neurons.

These endogenous plant compounds that act as anesthetics on animals are only released after the cell is ruptured and no longer living. Adjacent living cells are protected from these toxic compounds by cell walls or callose deposition in plasmodesmata in wounded cells to prevent symplastic movement (Faulkner, 2018). Thus, plants cannot anesthetize themselves as Reber et al claim. Plant-derived anesthetics are merely part of the defense response to discourage further predator attack (Sirikantaramas et al, 2007). Their existence does not mean that they function in stress-relief or pain-relief, nor does it indicate plants are conscious.

## Discussion

As far as we know, consciousness requires multicellularity and a fairly complex nervous system. As yet, there is no evidence for consciousness in prokaryotes, sponges, plants or fungi. Rather than being simple machines, living organisms have evolved through natural selection as complex systems that respond adaptively to their environments via their genetic and epigenetic programming. According to the dominant view to which we adhere, such adaptive behavior can be either conscious or nonconscious, depending on the presence or absence of a nervous system and a brain of threshold complexity.

In contrast, the CBC theory claims that all cells are sentient and volitional; that is, capable of making conscious decisions. There is no need though to invoke consciousness, considering the well-documented success of experimental approaches to cell biology based on standard molecular genetic models. The cellular mechanisms for all known developmental phenomena involve multiple receptors coupled to overlapping signal-transduction pathways. The interactions of these pathways are necessary and sufficient to enable cells to sense their environments and respond adaptively.

The CBC model makes unproven assertions about biological evolution. First, there is no experimental support for the notion that cells consciously direct their own evolution, a claim that was stated as “Life is not propelled by evolution or natural selection [but by] the consciousness that resides in all living things” (p. 52 of the parent book). Second, CBC claims that the standard, neo-Darwinian model called the Modern Evolutionary Synthesis (MES) is wrong because it considers only random genetic mutations and natural selection and fails to consider epigenetic changes as the drivers of evolution. In other words, CBC says cells sense their environments and use the epigenetic responses to modify their genomes and thereby guide their own evolution. All this postulates a new kind of Lamarckian evolution, whereby natural selection no longer plays a part.

Actually, the possible evolutionary role of epigenetic changes is unclear, because it is uncertain whether they can be inherited over multiple generations in natural environments, and therefore whether they contribute to long-term evolutionary change (Sarkies, 2023). In any case, there is absolutely no evidence that epigenetic changes are consciously induced. Finally, CBC mischaracterizes current evolutionary thinking: MES is not failing but remains the dominant view (Futuyma and Kirkpatrick, 2022) while being open to new evidence from epigenetics (Skinner and Nilsson, 2021).

Other aspects of CBC are outside the biological mainstream and lack empirical support. CBC says, “eukaryotic cells are multicellular organisms (cells within cells),” with an animal cell derived from three initially independent prokaryote cells—all of which were conscious—and a plant cell derived from four such cells (Baluška and Reber 2019). CBC also claims that each cell in a multicellular, collaborating group has a *theory of mind*, which is one of the highest-order aspects of consciousness (see “Definitions” in Box 1), for which experimental evidence exists only for humans, apes, monkeys, dolphins and crows. This speculative claim is stated as, “to collaborate in their trillions, each self-referential cell must “know that it knows,” “know that other cells know,” …” (Reber et al, 2023, p. 4). Such an assertion begs the question, how can a cell know something that most large-brained mammals cannot?

Reber et al (2023) portray CBC as an alternative theory that solves the main problems with standard theories of consciousness by bridging major disagreements between these theories, avoiding human-centered bias, and pinpointing the time when consciousness first evolved. However, an alternative theory cannot convince just because it seems helpful, convenient or “fresh” (p. 1). There is nothing wrong with CBC formulating testable hypotheses in a Popperian approach. But to become accepted, theories require proof from hypotheses-testing, solid facts and empirical evidence. In these respects, CBC falls short. Far from heralding in “a paradigm shift in evolutionary biology,” the CBC theory represents merely an intellectual exercise without empirical evidence that becomes less convincing the more it is repeated.

For further readings on the topics in this article, see Box 2.

But to become accepted, theories require proof from hypotheses-testing, solid facts and empirical evidence.

Box 2 Selected references for further reading **Consciousness**

Andelman-Gur MM, Fried I (2023) Consciousness: a neurological perspective. Acta Neurochirurgica 165:1-7 10.1007/s00701-023-05738-9

Birch J, Schnell AK, Clayton NS (2020) Dimensions of animal consciousness. Trends in Cognitive Sciences, 24(10):789-801. 10.1016/j.tics.2020.07.007

Mallatt J, Robinson DG, Blatt MR, Draguhn A, Taiz L (2023) Plant sentience: The burden of proof. Animal Sentience 8(33):15. 10.51291/2377-7478.1802

Robinson DG, Draguhn A (2021) Plants have neither synapses nor a nervous system. Journal of Plant Physiology 263, 153467. 10.1016/j.jplph.2021.153467

**Plant “neurotransmitters”**

Gao YQ, Jimenez-Sandoval P, Tiwari S, Stolz S, Wang J, Glauser G, … Farmer EE (2023) Ricca’s factors as mobile proteinaceous effectors of electrical signaling. Cell 186(7):1337–1351. 10.1016/j.cell.2023.02.006

Li L, Dou N, Zhang H, Wu C (2021) The versatile GABA in plants. Plant Signaling & Behavior, 16(3), 1862565. 10.1080/15592324.2020.1862565

**Plant anesthetics**

Draguhn A, Mallatt JM, Robinson DG (2020) Anesthetics and plants: no brain, no pain, and therefore no consciousness. Protoplasma 258, 239–248. 10.1007/s00709-020-01550-9

Jakšová J, Rác M, Bokor B, Petřík I, Novák O, Reichelt M, Mithöfer A, Pavlovič A (2021) Anaesthetic diethyl ether impairs long-distance electrical and jasmonate signaling in *Arabidopsis thaliana*. Plant Physiology and Biochemistry, 169:311–321. 10.1016/j.plaphy.2021.11.019

Tsuchiya H (2017) Anesthetic agents of plant origin: a review of phytochemicals with anesthetic activity. Molecules 22, 1369; 10.3390/molecules22081369

### Supplementary information

Peer Review File
