# Supplementary material for: Cell consciousness: a dissenting opinion: The cellular basis of consciousness theory lacks empirical evidence for its claims that all cells have consciousness
Source: EMBO Rep. 2024 Mar 28;25(5):2162–7. doi: 10.1038/s44319-024-00127-4 (PMC11094104; doi:10.1038/s44319-024-00127-4)
Supplement: Supplementary file 1 — Peer Review File [file 44319_2024_127_MOESM1_ESM.pdf]

EMBO reports

Cell consciousness: A dissenting opinion

David Robinson, Jon Mallatt, Wendy Peer, Victor Sourjik, and Lincoln Taiz

Corresponding author(s): David Robinson (David.Robinson@urz.uni-heidelberg.de)

Review Timeline:

Submission Date:	13th Mar 24
Accepted:	19th Mar 24

Editor: Holger Breithaupt

Transaction Report: This article was editorially reviewed.
